# Supplementary material for: Tissue- and Temporal-Dependent Dynamics of Myeloablation in Response to Gemcitabine Chemotherapy
Source: Cells. 2024 Aug 7;13(16):1317. doi: 10.3390/cells13161317 (PMC11352862; doi:10.3390/cells13161317)
Supplement: Supplementary file 1 [file cells-13-01317-s001.zip › cells-3083696-supplementary Figure.pdf]

Figure S1.

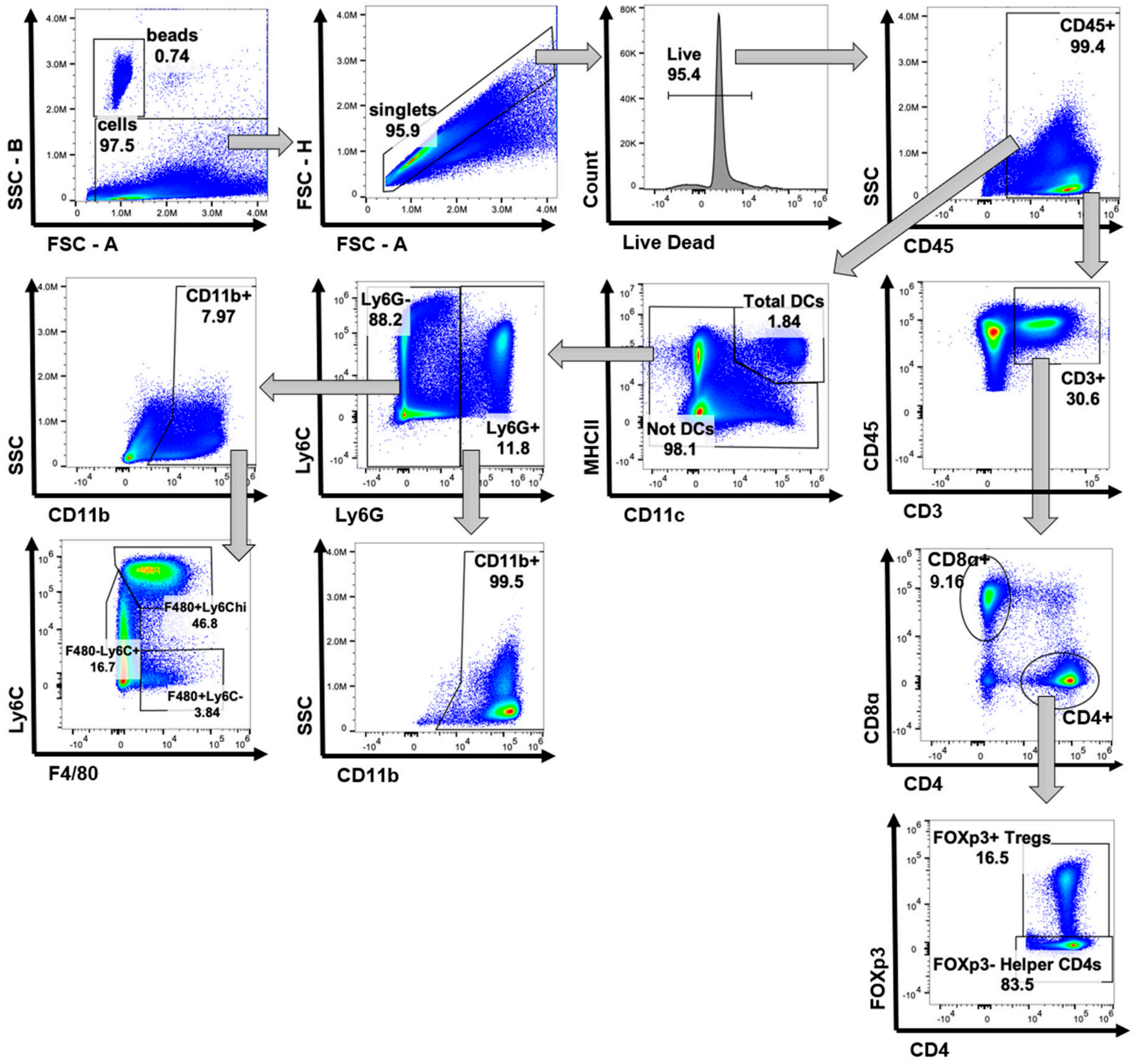

Supplement Figure 1. Gating strategy for spleen flow cytometry analysis in Sup. Fig 2, Sup. Fig 8 & Supp Fig 10. Gating strategy presented for the following immune cell subsets: dendritic cells (Live/CD45$^+$CD11c$^+$MHCII$^+$); granulocytes (Live/CD45$^+$Not DCs Ly6G$^+$CD11b$^+$); macrophages (Live/CD45$^+$Not DCs Ly6G$^-$CD11b$^+$F4/80$^+$Ly6C$^-$); inflammatory monocytes (Live/CD45$^+$Not DCs Ly6G$^-$CD11b$^+$F4/80$^+$Ly6C^{hi}); and monocytes (Live/CD45$^+$Not DCs Ly6G$^-$ CD11b$^+$F4/80$^-$Ly6C$^+$). CD8α T cells (Live/CD45$^+$CD3$^+$CD8α$^+$); CD4 helper T cells (Live/CD45$^+$CD3$^+$CD4$^+$FOXp3$^-$); regulatory T cells (T$_{regs}$; Live/CD45$^+$CD3$^+$CD4$^+$FOXp3$^+$); All frequencies shown are of the parent gate.

Figure S2.

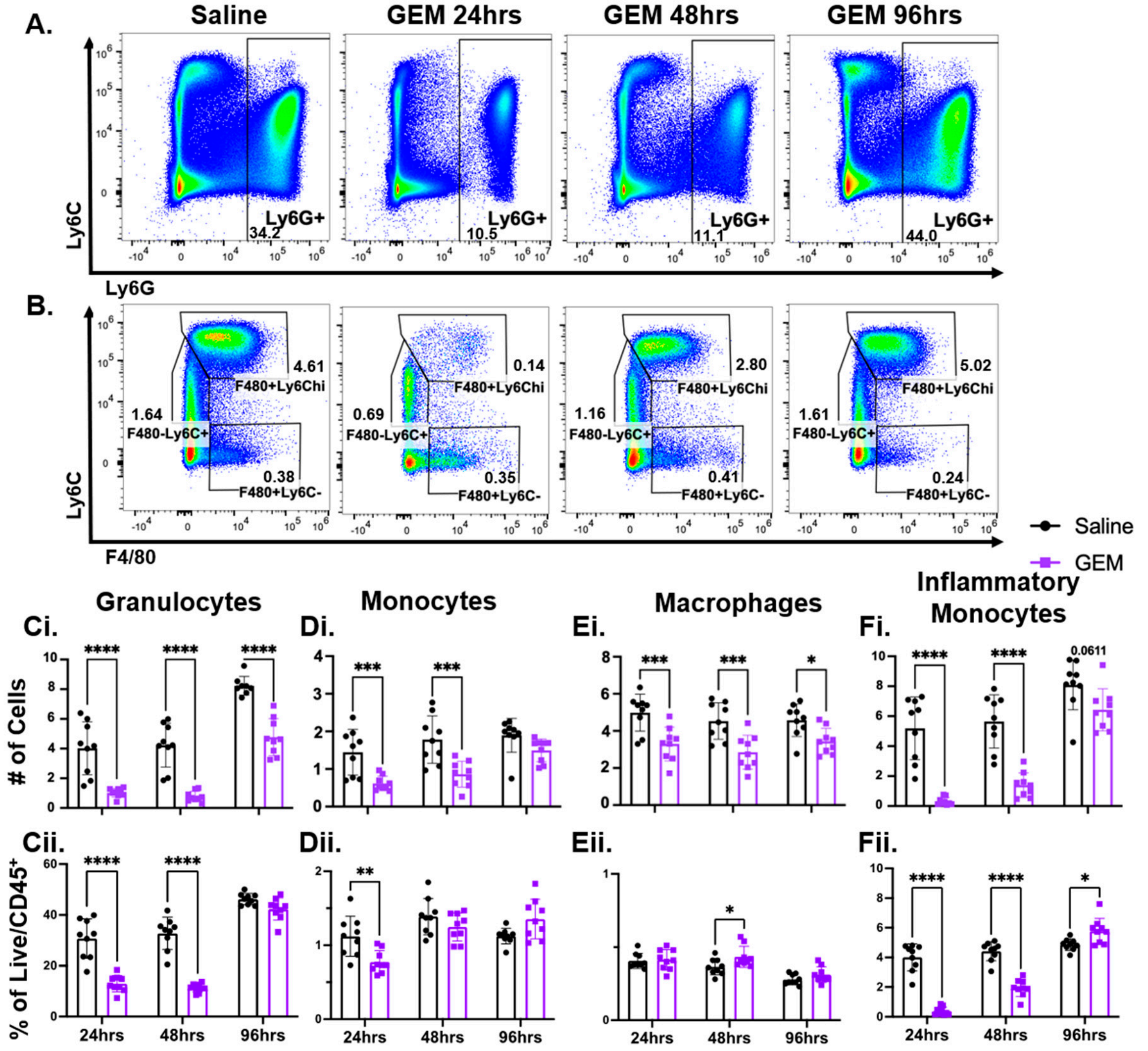

Supplemental Figure 2. Gemcitabine acts as a myeloablative chemotherapy systemically. 350k 4T1 parental cells were inoculated in the right flanks of BALB/c mice. Mice were injected with GEM (1.2mg) or saline I.P. on day 14 post inoculation. Spleens were excised 24, 48 and 96hrs post injection **A.** Representative scatter flow plots showing changes in the granulocyte ($CD11b^+Ly6G^+$) population post GEM administration. **B.** Representative flow plots of inflammatory monocytes ($CD11b^+Ly6G^-F4/80^+Ly6C^{hi}$), monocytes ($CD11b^+Ly6G^-F4/80^-Ly6C^+$) and macrophages ($CD11b^+Ly6G^-F4/80^+Ly6C^-$) post GEM injection. All frequencies shown are of the Live/$CD45^+$ population. **C.** Changes in granulocyte number on the order of 10^7 (**Ci**) and proportion (**Cii**) 24, 48 and 96hrs post injection. **D.** Changes in monocytes number on the order of 10^6 (**Di**) and proportion (**Dii**). **E.** Changes in macrophages number on the order of 10^5 (**Ei**) and proportion (**Eii**). **F.** Changes in inflammatory monocytes number on the order of 10^6 (**Fi**) and proportion (**Fii**).(n=9) (2way ANOVA with multiple comparisons: * $P<0.05$, ** $P<0.01$, *** $P<0.001$, **** $P<0.0001$; ROUT Outliers analysis with Q=0.1%). All points represent mean ± SD.

Figure S3.

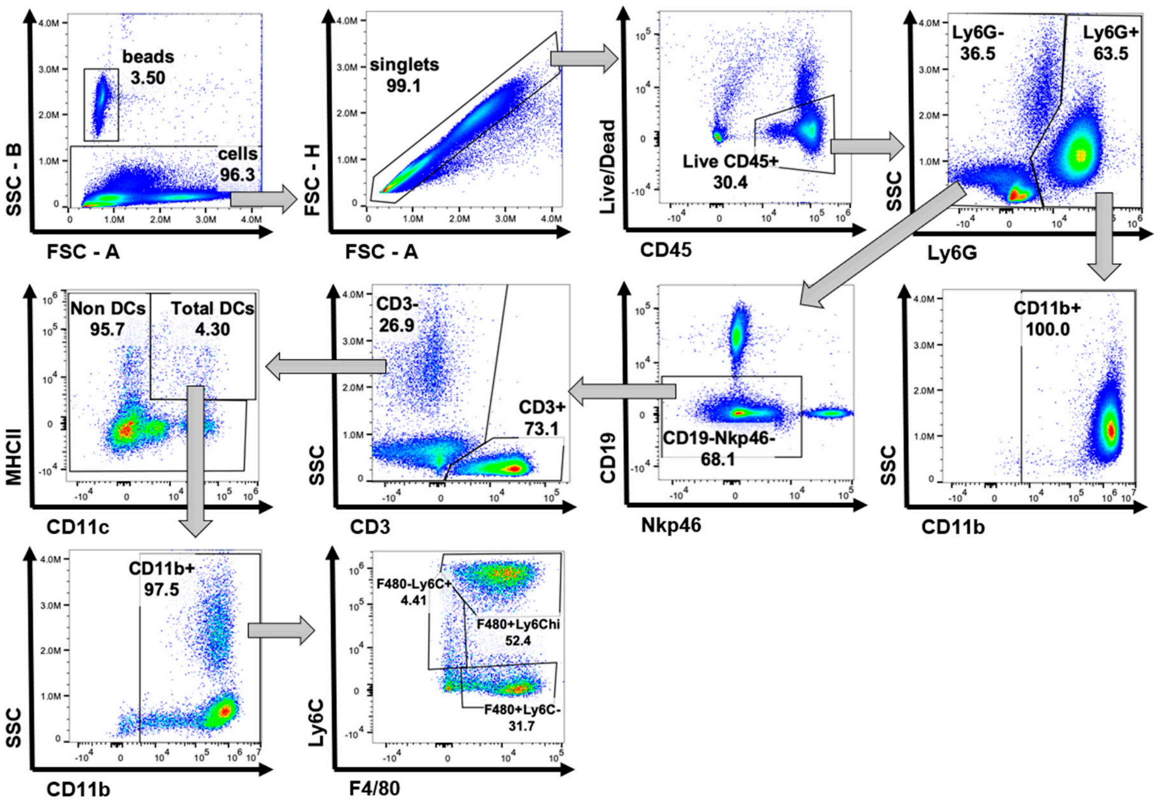

Supplement Figure 3. Gating strategy for blood flow analysis in Sup. Fig 4. Gating strategy presented for the following immune cell subsets: granulocytes (Live/CD45$^+$Ly6G$^+$CD11b$^+$); monocytes (Live/CD45$^+$Ly6G$^-$CD19$^-$Nkp46$^-$CD3$^-$Non DCs CD11b$^+$F4/80$^-$Ly6C$^+$); macrophages (Live/CD45$^+$Ly6G$^-$CD19$^-$Nkp46$^-$CD3$^-$Non DCs CD11b$^+$F4/80$^+$Ly6C$^-$); inflammatory monocytes (Live/CD45$^+$Ly6G$^-$CD19$^-$Nkp46$^-$CD3$^-$Non DCs CD11b$^+$F4/80$^-$Ly6C^{hi}); dendritic cells (Live/CD45$^+$Ly6G$^-$CD19$^-$Nkp46$^-$CD3$^-$CD11c$^+$MHCII$^+$); T cells (Live/CD45$^+$Ly6G$^-$CD19$^-$Nkp46$^-$CD3$^+$); All frequencies shown are of the parent gate.

Figure S4.

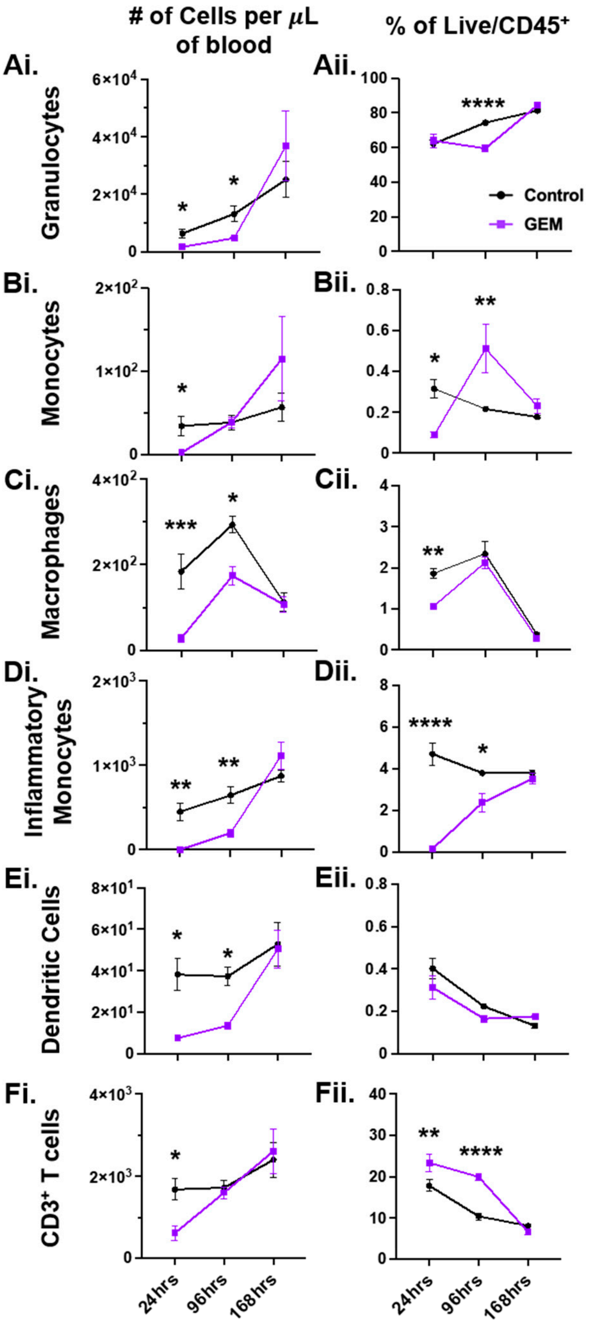

Supplemental Figure 4. Gemcitabine acts as a myeloablative chemotherapy on circulating immune cells. 350k 4T1 parental cells were inoculated in the right flanks of BALB/c mice. Mice were injected with GEM (1.2mg) or control IgG (200μg) I.P. on day 14 post inoculation. Blood was collected 24, 96 and 168hrs (7 days) post GEM. **A.** Changes in granulocyte ($CD11b^+Ly6G^+$) number per μL of blood (**Ai**) and proportion (**Aii**) following GEM administration. **B.** Changes in monocyte ($CD11b^+Ly6G^-F4/80^-Ly6C^+$) number (**Bi**) and proportion (**Bii**). **C.** Changes in macrophage ($CD11b^+Ly6G^-F4/80^+Ly6C^-$) number (**Ci**) and proportion (**Cii**). **D.** Changes in inflammatory monocyte ($CD11b^+Ly6G^-F4/80^+Ly6C^{hi}$) number (**Di**) and proportion (**Dii**). **E.** Changes in dendritic cell ($CD11c^+MHCII^+$) number (**Ei**) and proportion (**Eii**). **F.** Changes in circulating $CD3^+$ T cell number (**Fi**) and proportion (**Fii**) following GEM administration. All frequencies shown are of the Live/$CD45^+$ population. (n=5) (2way ANOVA with multiple comparisons: * P<0.05, ** P<0.01, *** P<0.001, **** P<0.0001; ROUT Outliers analysis with Q=0.1%). All points represent mean ± SEM.

Figure S5.

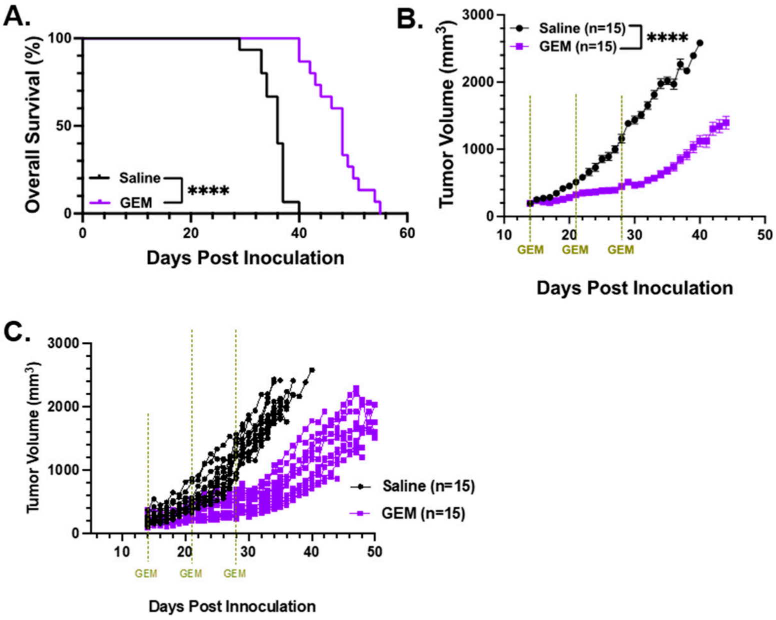

Supplemental Figure 5. Gemcitabine constrains 4T1 tumor outgrowth and significantly improves median survival in tumor-bearing mice. 350k 4T1 parental cells were inoculated in the right flanks of BALB/c mice. Mice were injected with GEM (1.2mg) or saline I.P. on days 14, 21 and 28 post inoculation. **A.** Kaplan-Meier curve depicting overall survival (significance assessed by log-rank (Mantel-Cox) test: **** P<0.0001). **B.** Average tumor growth curves for mice treated with GEM or Saline control. (Mixed-effects model: **** P<0.0001). All points represent mean ± SEM. **C.** Individual tumor growth curves of GEM or Saline treated mice.

Figure S6.

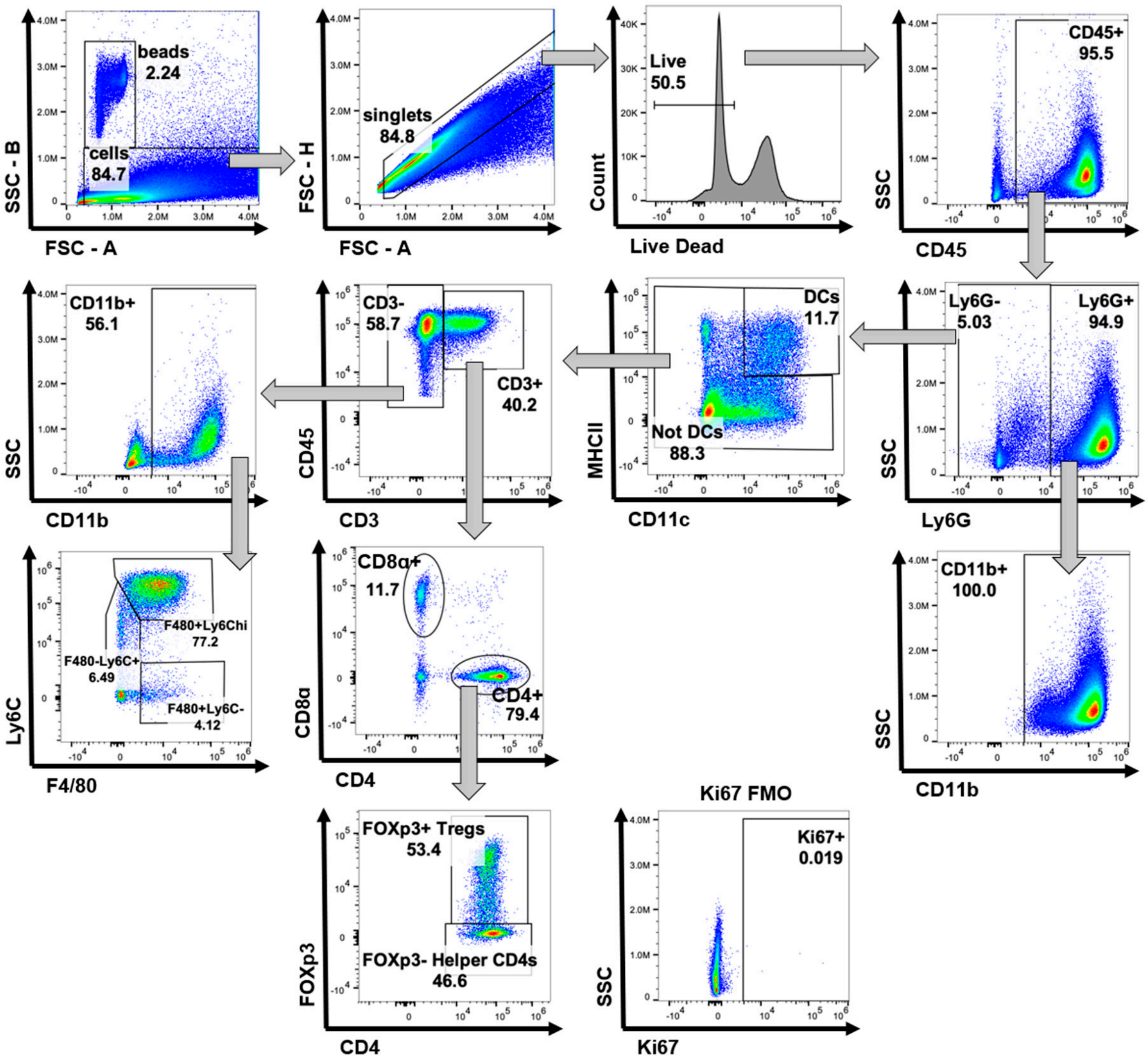

Supplement Figure 6. Gating strategy for tumor flow cytometry analysis in Fig 1, Fig 2, Fig 4 & Supp Fig 7. Gating strategy presented for the following immune cell subsets: granulocytes (Live/CD45$^+$Ly6G$^+$CD11b$^+$); dendritic cells (Live/CD45$^+$ Ly6G$^-$CD11c$^+$MHCII$^+$); CD8α T cells (Live/CD45$^+$Ly6G$^-$Not DCs CD3$^+$CD8α$^+$); CD4 helper T cells (Live/CD45$^+$Ly6G$^-$Not DCs CD3$^+$CD4$^+$FOXp3$^-$); regulatory T cells (T_{regs}; Live/CD45$^+$Ly6G$^-$Not DCs CD3$^+$CD4$^+$FOXp3$^+$) macrophages (Live/CD45$^+$Ly6G$^-$Not DCs CD3$^-$CD11b$^+$F4/80$^+$Ly6C$^-$); inflammatory monocytes (Live/CD45$^+$Ly6G$^-$Not DCs CD3$^-$CD11b$^+$F4/80$^+$Ly6C^{hi}); and monocytes (Live/CD45$^+$Ly6G$^-$Not DCs CD3$^-$CD11b$^+$F4/80$^-$Ly6C$^+$). All frequencies shown are of the parent gate.

Figure S7.

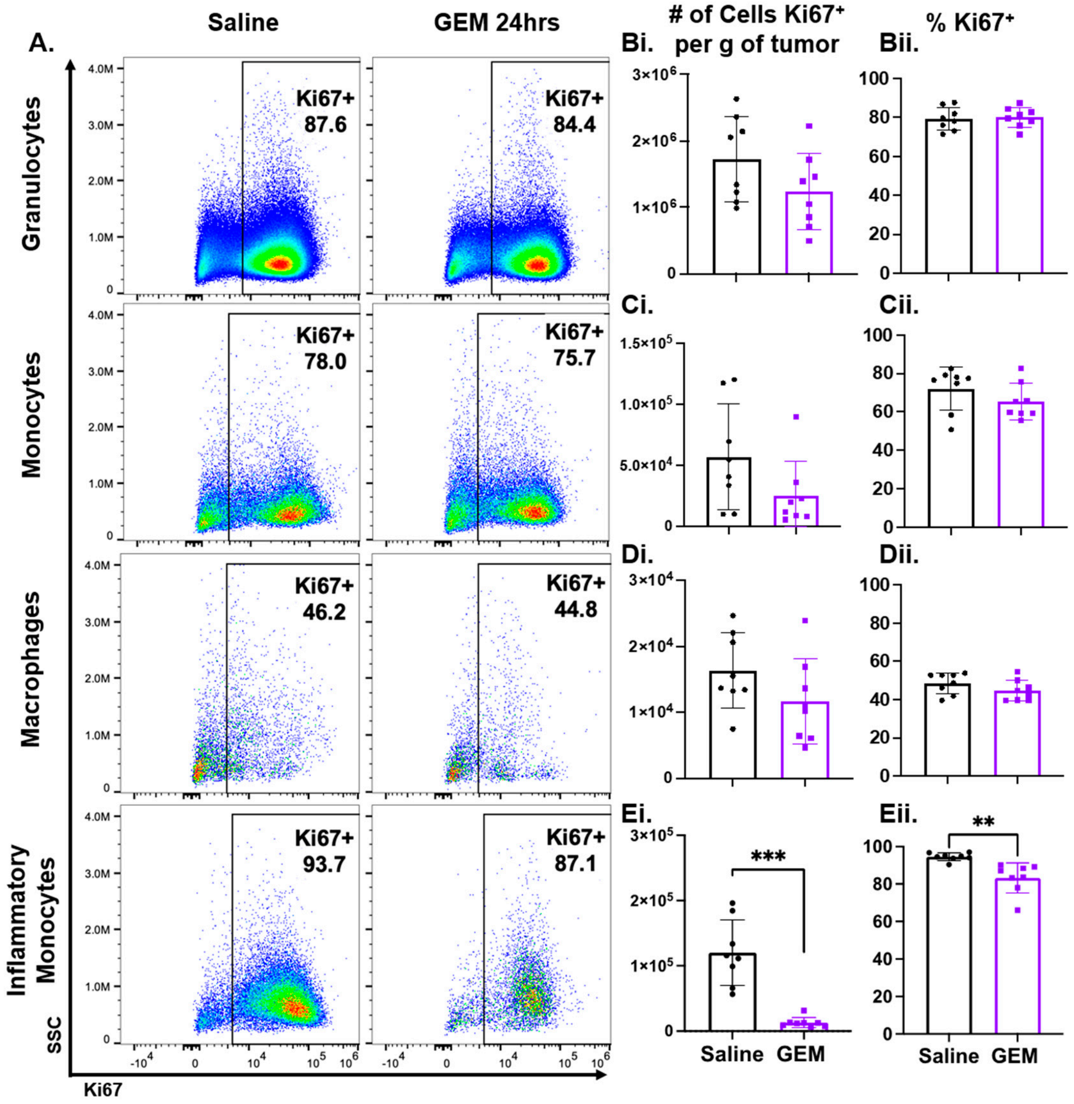

Supplemental Figure 7. Proliferation status of tumoral myeloid subsets & the effects of GEM on the Ki67⁺ cells. 350k 4T1 parental cells were inoculated in the right flanks of BALB/c mice. Mice were injected with GEM (1.2mg) or saline I.P. on day 14 post inoculation. Tumors were excised 24 post injection **A.** Representative scatter flow plots showing Ki67⁺ granulocytes, monocytes, macrophages and inflammatory monocytes in saline and GEM treated tumors. All frequencies shown are of the parent population. **B.** Changes in Ki67⁺ granulocyte number (**Bi**) and proportion Ki67⁺ (**Bii**). **C.** Changes in Ki67⁺ monocyte number (**Ci**) and proportion Ki67⁺ (**Cii**). **D.** Changes in Ki67⁺ macrophage number (**Di**) and proportion Ki67⁺ (**Dii**). **E.** Changes in Ki67⁺ inflammatory monocyte number (**Ei**) and proportion Ki67⁺ (**Eii**). (n=8) (Unpaired t test with Welch's correction: * $P<0.05$, ** $P<0.01$, *** $P<0.001$; ROUT Outliers analysis with Q=0.1%). All points represent mean ± SD.

Figure S8.

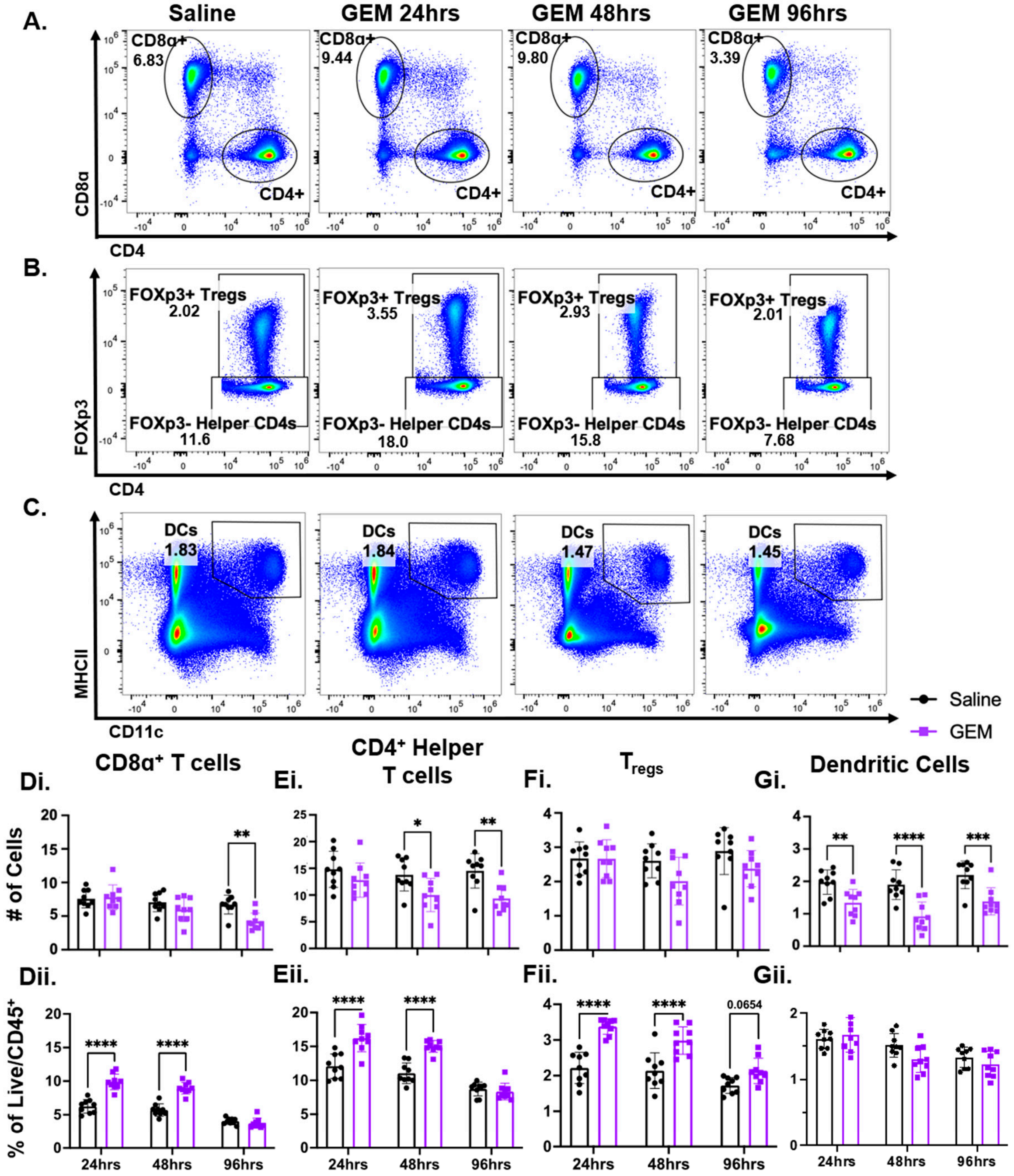

Supplemental Figure 8. The systemic effects of GEM on T cells and dendritic cells are less pronounced than what is observed in the TME. 350k 4T1 parental cells were inoculated in the right flanks of BALB/c mice. Mice were injected with GEM (1.2mg) or saline I.P. on day 14 post inoculation. Spleens were excised 24, 48 and 96hrs post injection **A.** Representative flow plots of CD8α$^+$ T cells (Ly6G$^-$Not DCs CD3$^+$CD8α$^+$) post GEM injection. **B.** Representative flow plots of CD4$^+$ helper T cells (Ly6G$^-$Not DCs CD3$^+$CD4$^+$FOXp3$^-$) and regulatory T cells (T_{regs}; Ly6G$^-$Not DCs CD3$^+$CD4$^+$FOXp3$^+$). **C.** Representative flow plots showing changes in dendritic cells (Ly6G$^-$ CD11c$^+$MHCII$^+$) post GEM administration. All frequencies shown are of the Live/CD45$^+$ population. **D.** Changes in CD8α$^+$ T cell number on the order of 10^6 (**Di**) and proportion (**Dii**). **E.** Changes in CD4$^+$ helper T cell number on the order of 10^6 (**Ei**) and proportion (**Eii**). **F.** Changes in T_{reg} number on the order of 10^6 (**Fi**) and proportion (**Fii**). **G.** Changes in dendritic cell number on the order of 10^6 (**Gi**) and proportion (**Gii**).). (n=9) (2way ANOVA with multiple comparisons: ⁎ P<0.05, ⁎⁎ P<0.01, ⁎⁎⁎ P<0.001, ⁎⁎⁎⁎ P<0.0001; ROUT Outliers analysis with Q=0.1%). All points represent mean ± SD.

Figure S9.

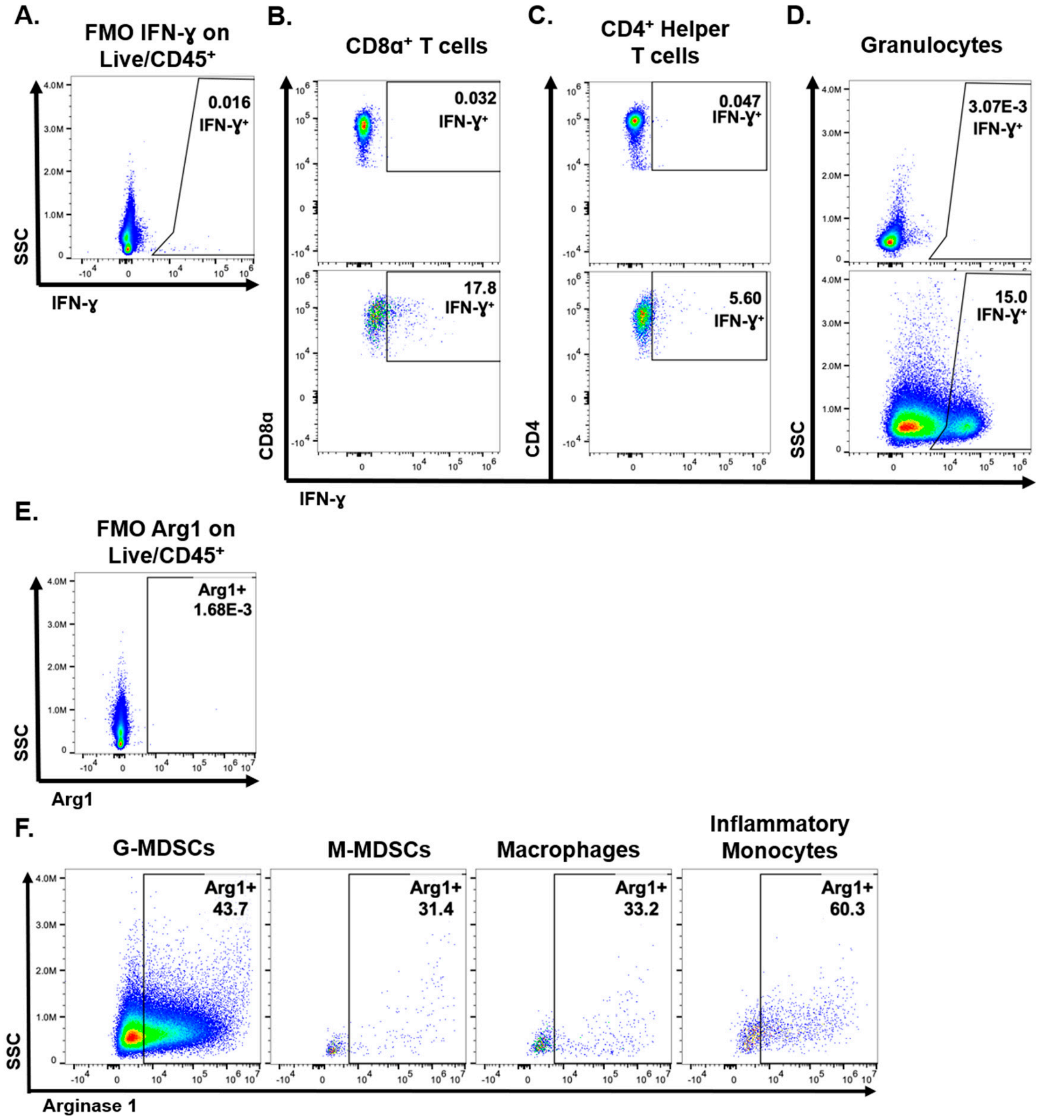

Supplemental Figure 9. Gates for tumor flow cytometry analysis in Fig 4, Fig 6 & Fig S10. A. Fluorescent minus one (FMO) staining control confirming the absence of IFN-ɣ+ signal on Live/CD45+ cells when the anti-IFN-ɣ fluorescent antibody is not present. **B.** Flow plots depicting the IFN-ɣ FMO on CD8α+ T cells (top) and a representative plot of IFN-ɣ+ CD8α+ T cells in the TME (bottom). **C.** Flow plots depicting the IFN-ɣ FMO on CD4+ helper T cells (top) and a representative plot of IFN-ɣ+ CD4+ helper T cells in the TME (bottom). **D.** Flow plots depicting the IFN-ɣ FMO on granulocytes (top) and a representative plot of IFN-ɣ+ granulocytes in the TME (bottom). **E.** FMO staining control confirming the absence of Arg1+ signal on Live/CD45+ cells when the anti-Arg1 fluorescent antibody is not present. **F.** Representative scatter flow plots of Arg1+ G-MDSCs, M-MDSCs, macrophages & inflammatory monocytes, respectively.

Figure S10.

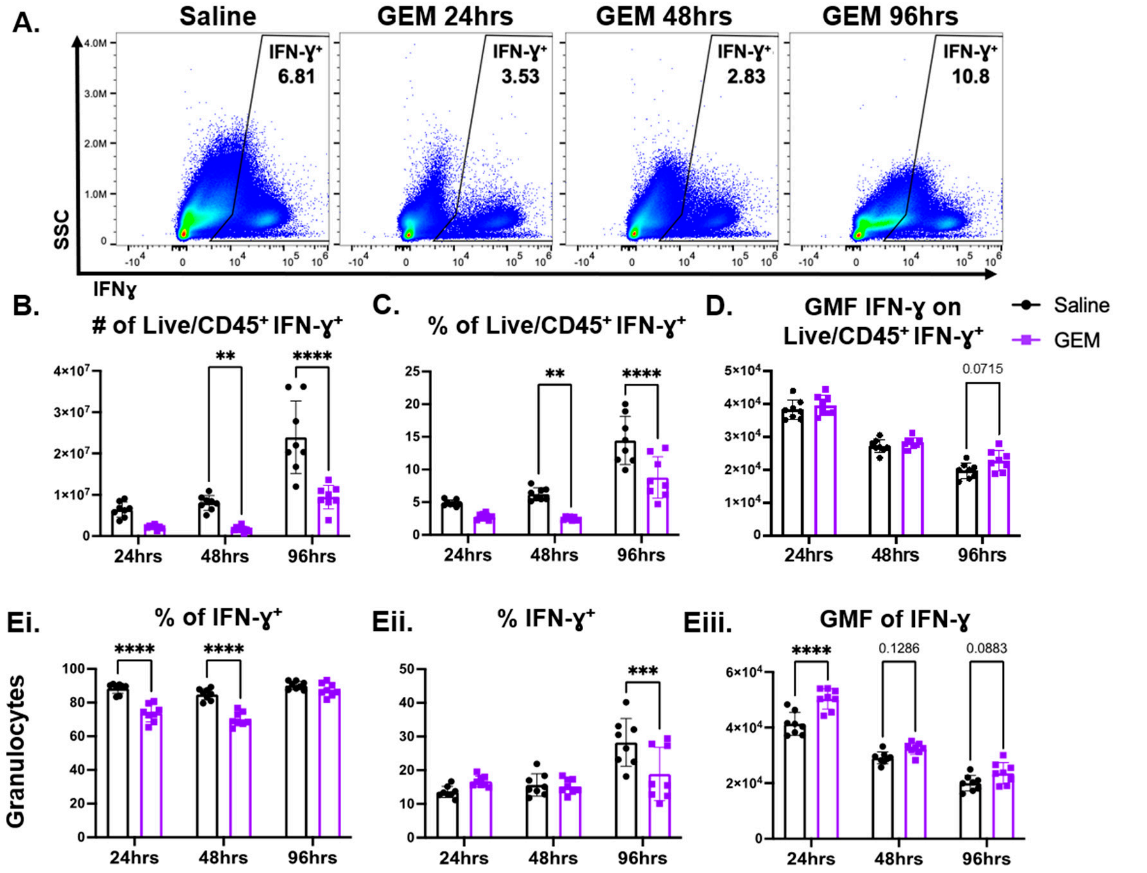

Supplemental Figure 10. GEM-induced effects on IFN-γ production in spleens of TNBC tumor-bearing mice. 350k 4T1 parental cells were inoculated in the right flanks of BALB/c mice. Mice were injected with GEM (1.2mg) or saline I.P. on day 14 post inoculation. Spleens were excised 24, 48 and 96hrs post injection **A.** Representative flow plots showing changes in the Live/CD45+ IFN-γ+ population following GEM administration. All frequencies shown are of the parent gate. **B.** Change in Live/CD45+ IFN-γ+ cell number. **C.** Change in the proportion of Live/CD45+ cells that are IFN-γ+. **D.** Change in Geometric Mean Fluorescence Intensity (GMF) of IFN-γ on Live/CD45+ IFN-γ+ cells. **E.** Changes in the proportion of IFN-γ+ cells that are granulocytes (**Ei**), the proportion of granulocytes that are IFN-γ+ (**Eii**), and the GMF of IFN-γ on IFN-γ+ granulocytes (**Eiii**). (n=8) (2way ANOVA with multiple comparisons: * $P<0.05$, ** $P<0.01$, *** $P<0.001$, **** $P<0.0001$; ROUT Outliers analysis with Q=0.1%). All points represent mean ± SD.
