# Supplementary material for: Tissue- and Temporal-Dependent Dynamics of Myeloablation in Response to Gemcitabine Chemotherapy
Source: Cells. 2024 Aug 7;13(16):1317. doi: 10.3390/cells13161317 (PMC11352862; doi:10.3390/cells13161317)
Supplement: Supplementary file 1 [file cells-13-01317-s001.zip › cells-3083696-supplementary Table.pdf]

Table S1. Key Resources Table

REAGENTS	SOURCE	IDENTIFIER
Anti-mouse CD45 clone 30-F11 BUV395	BD	Cat# 564279; RRID: AB_2651134
Anti-mouse CD3 clone 145-2C11 BUV563	BD	Cat# 749277; RRID: AB_287352
Anti-mouse MHCII clone M5/114 BUV496	BD	Cat# 750281; RRID: AB_2874472
Anti-mouse Ly6C clone HK1.4 BUV737	BD	Cat# 755201;
Anti-mouse CD8α clone 5H10-1 BUV805	BD	Cat# 752640;
Anti-mouse CD19 clone 1D3 BUV737	BD	Cat# 612781; RRID: AB_2870111
Anti-mouse CD3 clone 17A2 BUV737	BD	Cat# 564380; RRID: AB_2738781
Anti-mouse MHCII clone M5/114.15.2 AF647	BD	Cat# 562367; RRID: AB_11152078
Anti-mouse F4/80 clone BM8 BV421	BioLegend	Cat# 123137; RRID: AB_2563102
Anti-mouse Ly6G clone 1A8 BV605	BioLegend	Cat# 127639; RRID: AB_2565880
Anti-mouse Ly6C clone HK1.4 BV421	BioLegend	Cat# 128031; RRID: AB_2562177
Anti-mouse IFN- γ clone XMG1.2 PE/Dazzle 594	BioLegend	Cat# 505846; RRID: AB_505845
Anti-mouse Arginase 1 clone A1exF5 PE/Cy7	Invitrogen	Cat# 25-3697-82; RRID: AB_2734841
Anti-mouse/human CD11b clone M1/70 PE/Cy5	BioLegend	Cat# 101209; RRID: AB_312792
Anti-mouse Ly-6C clone HK1.4 PE/Dazzle 594	BioLegend	Cat# 128044; RRID: AB_2566577
Anti-mouse Ly6G clone 1A8 Alexa Fluor 647	BioLegend	Cat# 127610; RRID: AB_1134159
Anti-mouse CD335 (Nkp46) clone 29A1.4 PE	Invitrogen	Cat# 12-3351-82; RRID: AB_1210743
Anti-mouse CD11c clone N418 PE	Invitrogen	Cat# 12-0114-82; RRID: AB_465552
Anti-mouse Ki-67 clone SolA15 PerCP-eF 710	Invitrogen	Cat# 46-5698-80; RRID: AB_11039489
Anti-mouse CD3 clone 17A2 APC	Invitrogen	Cat# 17-0032-82; RRID: AB_10597589
Anti-mouse FOXp3 clone FJK-16s PE/Cy5	Invitrogen	Cat# 15-5773-82; RRID: AB_468806
Anti-mouse F4/80 clone BM8 Super Bright 780	Invitrogen	Cat# 78-4801-82; RRID: AB_2802477
Anti-mouse/human CD11b clone M1/70 FITC	Invitrogen	Cat# 11-0112-82; RRID: AB_464935
Anti-mouse CD4 clone RM4-5 eF450	Invitrogen	Cat# 48-0042-82; RRID: AB_1272194
Anti-mouse CD11c clone N418 eF450	Invitrogen	Cat# 48-0114-82; RRID: AB_1548654
Anti-mouse MHCII clone M5/114.15.2 AF700	Invitrogen	Cat# 56-5321-80; RRID: AB_494010
Fixable Live/Dead Aqua	Invitrogen	Cat# L34966; 405nm Excitation
Fixable Live/Dead Blue	Invitrogen	Cat# L23105; UV Excitation
Fc Block	Invitrogen	Cat# 14-0161-86; RRID: AB_467135
Brilliant Stain Buffer	BD	Cat# 563794
FACS Lysis	BD	Cat# 349202
